# A note on the laboratory culture of the benthic foraminifer *Cornuloculina balkwilli* (MacFadyen)

**DOI:** 10.1186/1756-0500-6-369

**Published:** 2013-09-11

**Authors:** Heidi A Seears, Christopher M Wade

**Affiliations:** 1 School of Biology, University of Nottingham, Nottingham, UK

**Keywords:** Benthic Foraminifera, *Cornuloculina balkwilli*, Laboratory culture, Asexual reproduction

## Abstract

**Background:**

Genetic studies of the Foraminifera provide valuable insights into marine speciation and biogeography, yet the discovery of vitally needed new genetic markers for this important group is being severely limited by an extreme lack of genetic data. The establishment of a laboratory culture from a single, asexually reproducing foraminifer, will be essential to provide enough pooled genetic material from these unicellular organisms, to facilitate full genome sequencing and genetic marker discovery, using next-generation sequencing techniques.

**Findings:**

The aim of this study was to develop a simple and inexpensive method of culturing benthic foraminifera, via asexual reproduction, in a controlled laboratory environment. Individual specimens of the benthic foraminfer *Cornuloculina balkwilli* (MacFadyen) were placed in 7 cm plastic beakers, containing 50 ml natural seawater, filtered to 0.2 μm, and kept at 23°C, with a 12-hour light/dark cycle, and fed weekly on a mixed algal diet of *Dunaliella tertiolecta* and *Phaeodactylum tricornutum.* Asexually derived cultures were successfully established from 4 specimens of *Cornuloculina balkwilli*, originally added to the culture vessels as immature specimens. Many thousands of individuals were present after 6 months.

**Conclusions:**

The method presented here demonstrates that only basic laboratory equipment is required to establish and maintain a thriving culture of the benthic foraminfer, *C. balkwilli*, from a single asexually reproducing specimen, providing an excellent source of genetic material for use in next generation sequencing. The method is easily reproducible and will greatly aid in the discovery of critically needed new genetic markers in the Foraminifera. It also highlights *C. balkwilli* as a good candidate species for use in the field of environmental micropaleontology.

## Findings

### Background

The Foraminifera are a diverse and widespread group of marine protists that are commonly used in genetic studies of marine speciation, biogeography, population genetics, and phylogenetics [1]. There is a pressing need to identify new genetic markers in the Foraminifera, to enhance both phylogenetic analyses and population genetic studies, however, our current ability to amplify and sequence new genes is severely limited by an extreme lack of genetic data for this important group. As foraminifera are unicellular, many individuals must be pooled to provide sufficient genetic material for full genome sequencing or for marker discovery via reduced representation techniques (e.g. S-RAD or RNA-Seq). Whilst specimens may be pooled from natural populations, minor genetic variation could lead to sequence ambiguities. Thus in order to obtain many identical copies of the genome it will be essential to establish a culture system in a controlled laboratory environment, starting from a single, asexually reproducing individual.

A number of species of benthic foraminifera have been successfully maintained (kept without reproduction) or cultured (grown and reproduced for several generations) under laboratory conditions [2-14]. Some culturing methods require sophisticated equipment, and provide a highly tailored environment [5,9,10], whilst others are simple in design, but easy to set up and less time-consuming to maintain [3-8,11-14].

Our aim was to culture a benthic foraminfer from a single asexually reproducing individual, using a simple culturing system. A basic culture vessel would be used (such as a petri dish or beaker), with light, temperature, and salinity manipulated manually using standard laboratory equipment. A variety of species of benthic foraminifera have been previously cultured, using only simple equipment, including *Allogromia laticollaris* (Arnold) [3], *Allogromia sp.*[7], *Spiroloculina hyaline* (Schulze) [4], *Rotaliella heterocaryotica* (Grell) [6], *Amphisorus hemprichii* (Ehrenberg) and *Amphistegina lobifera* (Larsen) [8], *Ammonia tepida* (Cushman) [11-13], and *Ammonia beccarii* (Linné) [13,14]. We selected a British intertidal benthic foraminfer that had not been previously cultured in the laboratory. *Cornuloculina balkwilli* (MacFadyen), of the suborder Miliolina, has a calcitic, porcellaneous, white test wall, giving it a slightly opaque appearance. The test is oval in outline, compressed, with each chamber forming roughly two thirds of a whorl, and the aperture is simple and terminal [15]. It is thought to be an inner shelf marine species, which is transported into the mouths of muddy estuaries [15], and is considered to be epifaunal, frequently attaching to available substrate, but also able to move freely [16]. The specific life cycle of *C. balkwilli* has yet to be documented, though as in most benthic foraminifera, it is likely to be dimorphic, consisting of a regular alternation between sexual and asexual generations [6,9,17-20]. It may even be trimorphic, involving the addition of successive asexual generations [9,13,21-27], which would be particularly favourable in terms of growing a large culture rapidly in the laboratory.

### Methods

#### Collection of specimens

**Figure 1:**
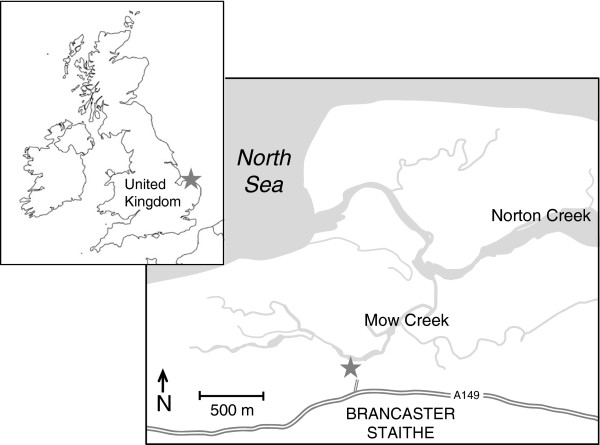
**Sampling location (indicated by a grey star) from which live specimens of the benthic foraminifer, *Cornuloculina balkwilli, *were collected.** The site is a tidal mudflat located in Mow Creek, at Brancaster Staithe, in North Norfolk, United Kingdom.

Live specimens of the benthic foraminfer *Cornuloculina balkwilli* (MacFadyen) were collected in May 2009, from tidal mudflats at Mow Creek, Brancaster Staithe, in Norfolk, UK (Figure 1). Samples were collected at low tide, from a 6 m^2^ site located approximately 1 – 2 m from the low-tide line. A thin layer of surface sediment was collected by hand (to a depth of ~ 1 cm), from areas where a greenish layer of algae (a food source for the foraminifera) was observed. The sediment was washed through a 106 μm sieve, with seawater collected from the same location, to concentrate the foraminifera and remove smaller particulate matter. Seawater to be used for the laboratory maintenance of the foraminifers was collected from the same location at high tide.

#### Laboratory culture

In the laboratory, 10 healthy specimens of *Cornuloculina balkwilli* (indicated by their bright orange cytoplasm and evidence of movement) were picked from the sediment, 5 of which were mature adults (~ 330 μm) and 5 of which were juveniles (~ 160 μm). Individual specimens were cleaned using a paintbrush (to remove bacteria and algae from the shell), before being placed in separate 7 cm plastic beakers containing 50 ml natural seawater, filtered to 0.2 μm as in [11,28], and covered with a loose lid to minimize evaporation. Using a 0.2 μm filter was necessary to prevent tiny gametes of the foraminifera (potentially carried in the seawater) from transferring through to the culture vessels, an important precaution if you wish to be certain that only asexual reproduction has occurred. Samples were kept at 23°C (ambient laboratory temperature), with lighting of 2 × 20w for twelve hours per day, regulated by an automated timer. Water salinity was maintained at between 27 – 30‰, within the natural range of the estuary collection site, with two thirds of the water volume replaced every 2 weeks. Specimens were fed weekly with a combination of the algal species *Dunaliella tertiolecta* (500 μl liquid culture) and *Phaeodactylum tricornutum* (5 drops liquid culture; Sciento, Manchester). Observations were carried out under a Nikon SMZ1500 light microscope after 3 and 6 months, and photographs taken using Nikon DXM1200F camera.

### Results and discussion

**Figure 2:**
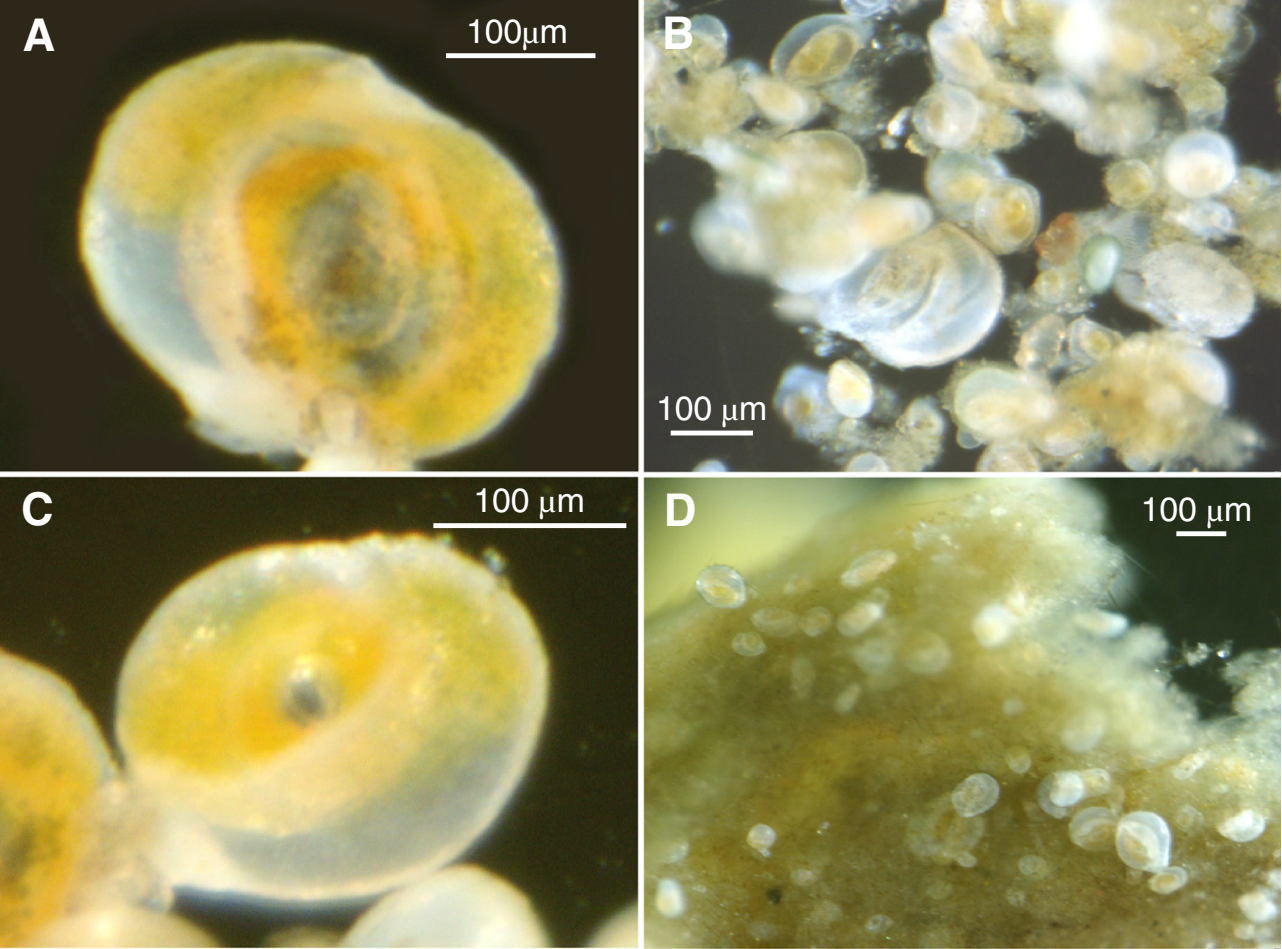
**Light microscope photographs of the benthic foraminifer *Cornuloculina balkwilli *in culture. A**, Adult specimen; **B**, Many different sized specimens from the culture population, indicating multiple reproductive events; **C**, large juvenile produced in culture; **D**, Juvenile specimens attached to an algal substrate.

The mature adult specimens of *Cornuloculina balkwilli* used in this study measured approximately 330 μm in size, with a proloculus of ~37 μm (Figure 2A). Observations of the mature adult specimens, taken after 3 months in the culture vessels, revealed that 3 out of 5 had died without reproducing, whilst the remaining 2 appeared healthy, but had also failed to reproduce. After 6 months, even these remaining 2 specimens had died, again with no evidence of reproduction.

Observations of the specimens originally added as juveniles, however, revealed evidence of reproduction from a single individual. The offspring observed were most likely produced via asexual reproduction, commonly seen in the foraminifera as part of a dimorphic or trimorphic life-cycle [17], although autogamy (self-fertilisation), which has been observed in some foraminifera [17], cannot be ruled out without further investigations.

After 3 months, thriving populations had established in 4 out of 5 culture vessels (the remaining specimen had died). Hundreds of individuals of many different sizes were present in the 4 successful cultures, indicating multiple reproductive events (Figure 2B). The smallest juveniles were approximately 60 – 70 μm in size, whilst some had grown up to ~220 μm (Figure 2C). The specimens had attached in large numbers to an algal substrate that had formed in the culture vessels (presumably from the *Dunaliella tertiolecta* and *Phaeodactylum tricornutum* added as food) (Figure 2D), and were heavily crowded, making it impossible to determine how many young were produced per brood. After 6 months the 4 successful cultures were all still thriving and reproducing, each containing many thousands of individuals.

It is interesting to note that only individuals that were added as immature specimens reproduced. It has been observed, in the benthic foraminifer *Ammonia tepida,* that any mature adults surviving after a reproductive event do not usually reproduce again [12]. It is possible that the large adult specimens of *C. balkwilli* collected from the field had already reproduced in their natural habitat, and would therefore not do so again, making it preferable to choose less mature specimens for culture experiments.

These experiments demonstrate that it is possible to successfully culture specimens of the benthic foraminfer *Cornuloculina balkwilli* from a single individual via asexual reproduction, and also show that only basic laboratory equipment is required, negating the need for sophisticated systems such as circulating and re-circulating marine aquaria and other flow-through systems or expensive commercially available chemostats. A continuously reproducing culture of *C. balkwilli* was obtained and will be maintained and ultimately used as a source of RNA and DNA for molecular marker discovery using next generation sequencing technologies. In addition, we propose that the simple culturing method used here, together with the species *C. balkwilli* would be ideally suited for use in environmental micropaleontological studies, for which laboratory cultures of other benthic foraminiferal species have already proven invaluable [11,29-34].

As a technical point, it should be noted that the DNA of the food organisms added to the culture system could become a source of contamination during subsequent molecular procedures. The genomic DNA of the food organisms would therefore need to be sequenced alongside that of the foraminifera, for comparison and elimination from further analysis. The same would apply if any symbionts or commensals were present, though this is not thought to be the case in this particular species. To minimize contamination from prey particles, all foraminifers should be kept without food for a period of time prior to the extraction of their DNA/RNA.

## Competing interests

The authors declare that they have no competing interests.

## Authors’ contributions

HAS and CMW conceived and designed the study. HAS and CMW conducted the fieldwork. HAS conducted the laboratory work. HAS and CMW analyzed data. HAS and CMW wrote the manuscript. Both authors read and approved the final manuscript.
